# COVID-19 Knowledge Test: An Assessment Tool for Health Educators During the COVID-19 Pandemic

**DOI:** 10.3389/fpubh.2020.580204

**Published:** 2020-11-05

**Authors:** Lindsy J. Richardson, Jocelyn J. Bélanger

**Affiliations:** ^1^College of Social and Behavioural Sciences, Walden University, Minneapolis, MN, United States; ^2^Department of Psychology, New York University, Abu Dhabi, United Arab Emirates

**Keywords:** COVID-19, knowledge, health education, test, scale development, Rasch analysis

## Abstract

**Background:** As of August 11, 2020, Coronavirus disease 2019 (COVID-19) has infected 19,936,210 persons and led to 732,499 deaths worldwide. The impact has been immense, and with no vaccine currently available, the best way to protect our communities is health education. We developed a brief COVID-19 knowledge test for health educators that can be used to assess deficits in clients' understanding of the disease.

**Methods:** COVID-19 Knowledge Test items were developed by the research team and administered to participants. An alternate-choice item format was selected for the knowledge test, and data analysis was based on an American sample of 273 respondents. A detailed analysis of the data was conducted with classical test theory and Rasch analysis.

**Findings:** The final instrument was found to be a unidimensional measure of COVID-19 knowledge. Results provided evidence for absolute model fit and model fit for individual items. All items included on the scale were monotonically increasing and split-half reliability was considered acceptable. Total test information revealed that the test is suitable for individuals with low to average knowledge of COVID-19.

**Interpretation:** Rasch analysis provides support for the COVID-19 Knowledge Test to be used as an assessment tool for health educators. The final version of the test consists of 34 high-quality test items that can be administered in <10 min. Normative data and suggested cutoff scores are also provided.

## Introduction

*Scientia potentia est*, the Latin phrase for knowledge is power, is the public's best defense against COVID-19, and knowledge of the disease is crucial to convincing people to take precautions, such as staying home, physically distancing, and following other preventative measures. According to Van den Broucke (1), health education is only effective in changing behavior when it meets four criteria: (a) Are they susceptible to the condition? (b) Would the condition be severe? (c) Is prevention effective? (d) Lastly, can the preventative actions be performed? With no vaccine currently available, it is essential for health educators to accurately assess public understanding, and then deliver education where need exists.

Coronavirus disease 2019 (COVID-19) emerged as a cluster of pneumonia cases in December 2019 in Wuhan China, and, as of August 11, 2020, there have been 19,936,210 confirmed cases and 732,499 deaths around the world (2). COVID-19 is highly transmissible. On average, infected individuals have been shown to infect up to three others. Evidence also suggests that asymptomatic people can transmit the virus (3). Additionally, the mortality rate of COVID-19 is significant even among otherwise healthy people and more dangerous to the elderly and other vulnerable populations. The fact that the disease kills otherwise healthy adults, in addition to elderly and other vulnerable populations, is a challenge for health care systems. COVID-19 has had a large impact on mental health (4, 5), and the spread of misinformation can lead to mistrust, panic, and misunderstandings about COVID-19 (6).

Timely public health education is crucial for the prevention of emerging and reemerging infectious diseases (7) and has been previously applied to improve the general population's understanding (8). Individuals with poor knowledge of prevention are less likely to adhere to medical instructions (9). Consequently, continued health education during the COVID-19 pandemic is recommended to ensure people understand the basic facts of the disease and to provide support for people in developing key behaviors to remain healthy.

To help health educators (e.g., physicians, social workers, psychologists, teachers, public health educators) apply effective interventions, we developed a short test that provides an accurate indication of a test taker's general knowledge of COVID-19. The COVID-19 knowledge test could be used prior to a learning intervention to gauge what their clients know and do not know about the current research and facts on COVID-19. Education programs can then be tailored toward various levels of learners instead of using a one size fits all approach.

The COVID-19 knowledge test was found to be a reliable unidimensional instrument that can be administered in under 10 min using Rasch measurement modeling. We incorporated a range of items that could discriminate between test takers with different levels of knowledge by including varying levels of difficulty. Item analysis is an important tool to ensure the quality of a test and to accumulate a bank of well-written items. It is also useful for identifying items that may be too easy or too difficult and that may fail to differentiate between individuals who are highly knowledgeable of COVID-19 and those with little knowledge.

Raw test scores can lead to errors in analyses when comparing test takers. An educator may be inclined to sum raw scores, but it is unlikely that all test items are equally difficult. Comparing test takers based on totaling raw scores does not provide meaningful and accurate comparisons of knowledge between test takers. Thus, we used the Rasch measurement model to compute respondent performances in a meaningful way. Rasch measurement allows the meaning of a test to be explained in terms of the test's items, allowing test administrators to use raw test scores to explain test taker performance on a linear scale that accounts for unequal difficulties across all the test items (10).

## Method

Ethics approval for this study was provided by New York University's Institutional Review Board (HRPP-2020-69). Participants were drawn from Mechanical Turk (MTurk), a crowdsourcing internet site that permits people to complete surveys for nominal compensation. Participants were identified by a unique identification number. MTurk qualification filters were specified to only include American participants with a minimum of a 90% positive rating on previous MTurk tasks.

Three hundred and forty-two responses were initially received. After data screening, 273 responses remained. Participants were removed if they did not respond as expected to the attention check items or if they did not complete more than 75% of the questions. The mean age of participants was 40.06 (*SD* = 13.15) years. One hundred and fifty-two men and 119 women participated in the study. The racial distribution included 218 Caucasians, 22 blacks, 21 Asians, one Native American, and 12 who identified as biracial or other race.

About 1% of participants reported they did not have a high school diploma, 7% had a high school diploma or GED, 13.9% reported they had some college but no diploma, 7% had an associate degree, 45% had an associate or undergraduate degree, and 25% had a graduate degree. The majority of participants' primary source of knowledge about COVID-19 was the internet (61.2%) and television (32.6%). Less than 1% of participants' primary sources of knowledge were friends, family members, medical journals, and work.

## Material

### Item Development

Forty-nine items were developed to tap basic knowledge of COVID-19 through consulting peer-reviewed journals and reputable websites (e.g., the World Health Organization, The Lancet, Microbiology, and Infection). Initial item content consisted of medical terminology related to COVID-19, symptoms of the virus, a brief history of corona viruses, risk factors, and pertinent findings from emerging research. Once the items were developed content validity was reviewed by a three-person expert panel (two physicians and a doctoral educated panel member in biochemistry) and revised accordingly.

Alternate-choice item format was selected instead of true or false or multiple-choice, because it offers a comparison between two choices. One of the advantages of the alternate-choice format is that more questions can be asked in a testing period, which can create a more reliable test than multiple-choice format (11, 12). Further, alternate-choice tests have been found to exhibit satisfactory psychometric properties in previous research (13–16).

### Attention Items

In addition to participants answering basic items that tapped their knowledge of COVID-19, they were asked three questions to confirm they were paying attention. The attention check items were adapted from the SPECTRA Indices of Pathology Scale's Infrequency Scale (17) and were as follows: “I have difficulty remembering if I went to elementary school,” “I have never seen a dog,” and “I am answering these questions truthfully.”

## Results

Classical test theory (CTT) analysis was conducted first using the item.exam (data, discrim = TRUE) command in the psychometric library in the R software for statistical computing. An initial review of the 49 items revealed that items C3, C15, and C18 had negative discrimination values and were therefore deleted. Items with negative discrimination indices are problematic because they indicate that high-performing participants tend to provide incorrect responses and low-performing participants provide correct responses (18).

Following CTT analysis, item response theory (IRT) analyses were completed for the remaining items. The assumption of unidimensionality was first assessed using the mirt library, which was developed to estimate multidimensional item response theory parameters in R (19)^.^ According to Hattie (20), spurious factors can occur in exploratory factor analysis with dichotomous item response data, which can lead to errors and incorrect conclusions about the dimensionality of data. Instead, exploratory factor analysis models are specified using the information maximum likelihood expectation maximization (EM) algorithm of Bock and Aitken (21).

One- and two-factor exploratory factor analysis (EFA) models were first specified. The statistically significant results (*p* < 0.05) of the likelihood ratio test, along with the values for the Akaike information criterion (AIC) information index, indicated that a two-factor model (AIC = 11408.45) fit the data better than a one-factor model (AIC = 11399.62).

Since the assumption of unidimensionality was initially violated, the multidimensionality of the data was further explored through examining the factor loadings of the factor analysis. Items C5, C7, C9, C13, C14, C16, and C23 were deleted because they were loading on a second factor. The models were tested again, and the one-factor model fit the data better than the two-factor model.

To assess the assumption of monotonicity, the Rasch measurement model was applied. The data were fit to the model setting the item discrimination parameter value to equal one for all items. This allowed us to differentiate among examinees with different levels of knowledge of COVID-19. The data did not meet the assumption of monotonicity for item C17 because the relationship between the latent trait and probability of item endorsement was not monotonically increasing. Consequently, this item was deleted.

To test whether the model fit for the individual items, the item.fit (test.rasch, simulate.p.value = TRUE) command was used. Significant results for item fit indicated the model did not accurately fit the responses for each item (22). The model fit for the individual items, except for items C8, C20, C38, and C46, which were significant at the 0.01 level; thus, these items were also removed. Further, absolute model fit was assessed using a bootstrap model of fit test with the GoF.rasch (test.rasch, B = 1,000) command. The results were not significant (*p* = 0.22), demonstrating that the Rasch model adequately fit the data. Unidimensionality was assessed for a final time. The factor loadings can be found in Table 1, and the comparison of factor models can be found in Table 2.

**Table 1:** Factor loadings for the 34-item knowledge test.

**Item**	**F1**
C1	0.49
C2	0.26
C4	0.22
C6	0.60
C10	0.72
C11	0.58
C12	0.43
C19	0.36
C21	0.47
C22	0.26
C24	0.46
C25	0.47
C26	0.62
C27	0.03
C28	0.54
C29	0.61
C30	0.49
C31	0.58
C32	0.27
C33	0.05
C34	0.59
C35	0.31
C36	0.16
C37	0.45
C39	0.87
C40	0.55
C41	0.57
C42	0.42
C43	0.19
C44	0.35
C45	0.79
C47	0.75
C48	0.60
C49	0.27

*Oblimin rotation using the information maximum likelihood expectation maximization (EM) algorithm*.

**Table 2:** Comparison of factor models.

**Factors**	**AIC**	**AICc**	**SABIC**	**HQ**	**BIC**
1	8279.493	8325.493	8309.326	8378.02	8524.938
2	8285.559	8406.050	8329.870	8431.899	8650.116

The sum of squared loadings for the model was 8.34, and the proportion of variance in the observed variables associated with the one factor accounted for 24.5% of the variance present in the items. Consequently, we can conclude that the assumption of unidimensionality was met for the 34-item knowledge test and that one factor underlies the responses to the knowledge items.

In order to precisely estimate item difficulty, the Rasch model was applied again. The data were fit to the model with the item-discrimination parameter value set to equal one for all items. Item difficulty values for the knowledge test ranged between −3.58 and 0.57. Item difficulty is the point on the item characteristic curve where the *S*-shaped curve has the steepest slope. Examinees must have greater knowledge to answer a difficult item correctly. Less knowledgeable test takers are likely to answer items incorrectly, with values >1.00, whereas examinees with less knowledge will have a moderate chance of answering items with values <-1.00 correctly. Item difficulty values between −1.00 and 1.00 are considered moderately difficult; items <-1.00 are easy, and items >1.00 are difficult (23). Thirteen of the knowledge-test items were moderately difficult and the remaining 21 items were easy. Split-half reliability was computed with Kuder–Richardson Formula 20 (KR-20). KR-20 was 0.70, demonstrating an acceptable level of internal consistency (24).

**Figure 1:**
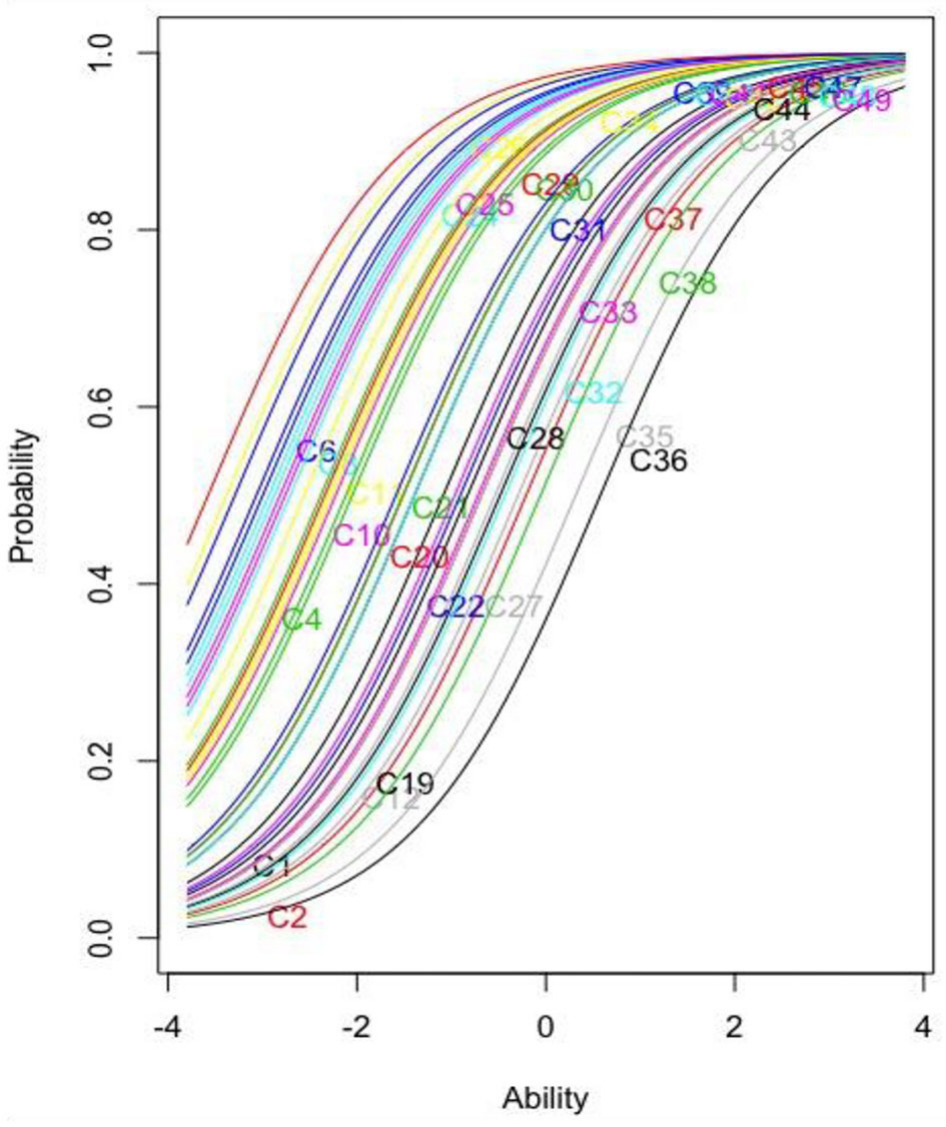
Item characteristics curve for the remaining 34 knowledge items.

Figure 1 presents the item characteristic curves for the remaining 34 items. The vertical axis displays the probability of success of a person on each item, ranging from 0.00 to 1.00. The horizontal axis displays a person's ability in log-odd units. When item difficulty and person ability are matched, the test taker has a 50% chance of success on that item (i.e., 50/50 odds). Item C2 is the closest item to 0.00 logits. Difficulty values for the remaining items can be found in Table 3. In addition, item *z*-values for the knowledge test items were all greater than two; *z*-values greater than two indicate that the item parameter is unlikely to be zero in the population (22).

**Table 3:** Item difficulty values, standard error, and *Z*-values.

**Item**	***b*-values**	**std.err**	***z*.vals**
C1	−1.09	0.16	−7.01
C2	−0.21	0.14	−1.49
C4	−2.39	0.21	−11.24
C6	−3.00	0.26	−11.39
C10	−2.23	0.20	−11.02
C11	−2.27	0.20	−11.08
C12	−0.54	0.15	−3.71
C19	−0.49	0.15	−3.36
C21	−1.52	0.17	−9.01
C22	−0.91	0.15	−6.01
C24	−2.88	0.25	−11.44
C25	−2.82	0.25	−11.45
C26	−3.38	0.31	−11.03
C27	−0.30	0.14	−2.07
C28	−0.84	0.15	−5.55
C29	−2.35	0.21	−11.19
C30	−2.12	0.20	−10.83
C31	−1.59	0.17	−9.30
C32	−0.44	0.15	−3.01
C33	−0.72	0.15	−4.87
C34	−2.56	0.23	−11.39
C35	0.31	0.14	2.17
C36	0.57	0.15	3.91
C37	−0.69	0.15	−4.64
C39	−3.30	0.30	−11.14
C40	−2.94	0.26	−11.42
C41	−2.76	0.24	−11.45
C42	−2.31	0.21	−11.14
C43	−0.69	0.15	−4.64
C44	−1.40	0.16	−8.50
C45	−3.58	0.33	−10.76
C47	−3.07	0.27	−11.35
C48	−1.40	0.16	−8.50
C49	−0.97	0.15	−6.35

*Akaike information criterion (AIC) = 8366.981, Bayesian information criterion = 8489.703, and log-likelihood value (logLik) = −4149.491*.

Figure 1 demonstrates the relationship between knowledge of COVID-19 and the probability of a correct response monotonically increases for the 34 knowledge items. This means that the more knowledge people have about COVID-19, the greater the probability of correctly answering an item. Relative difficulty can also be examined based on location in the graph. For example, item C45 is the easiest item because it is furthest to the left of the y-axis, while item C36 is the most difficult item since it is the furthest to the right.

**Figure 2:**
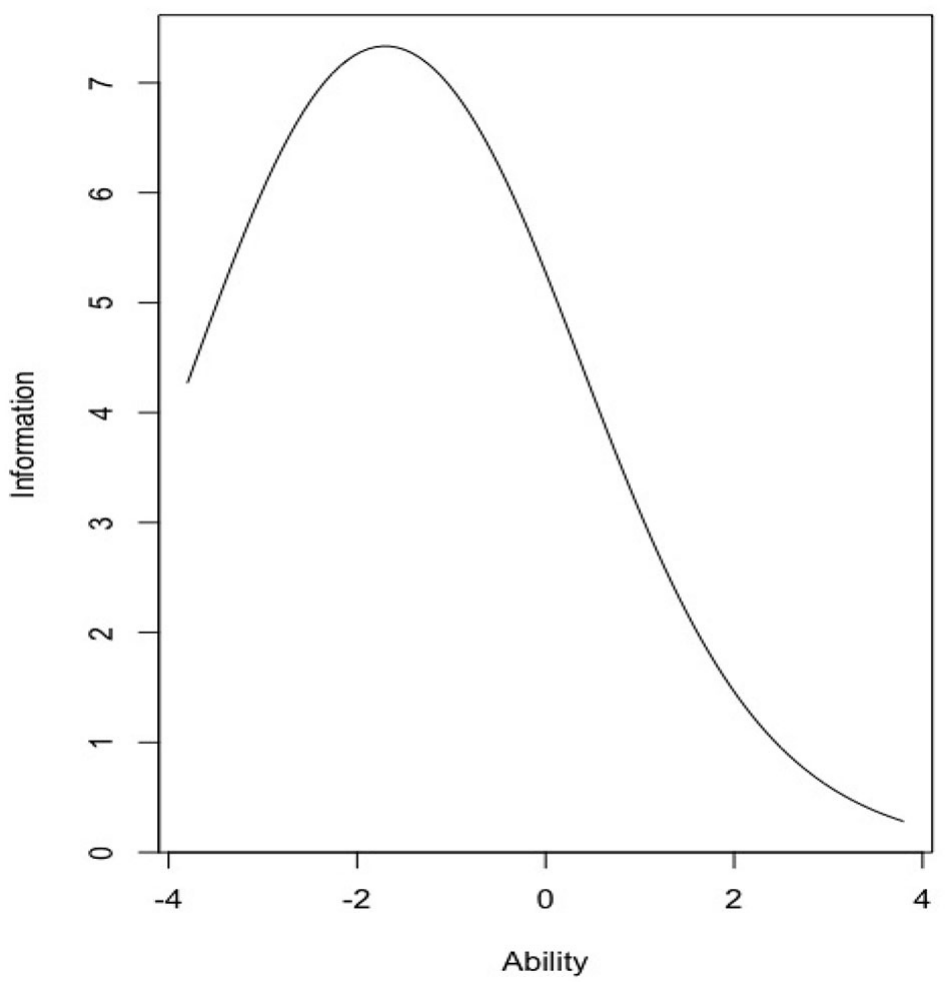
Total test information curve for the 34-item knowledge test.

The total test information curve (see Figure 2) demonstrates that maximum information for examinees was approximately −1.6 or slightly below average knowledge of COVID-19. Hence, this a good scale for discriminating between test takers who score in the −3.0 to +1.5 standard deviation range (i.e., very low when compared with average scores).

To understand the amount of information this instrument will provide for those with above average knowledge of COVID-19, a numeric estimate was obtained using the information (test.rasch, c[0,10]) command in R. In the above average range of knowledge, the total information yielded by the knowledge test was 33.99 or 20.96% of the total information provided by the Rasch measurement model. About 79.04% of the information is provided for knowledge levels below zero. Final item statistics can be reviewed in Table 3.

The total mean score for the remaining items was 26.27 (*SD* = 4.05). Based on a standard deviation of 4.05, scores below 21 are below average, scores between 22 and 29 are average, and scores 31 and higher are above average for this sample. Table 4 provides distributions of total score on the knowledge test by demographics. Overall, 15% of participants had a score below average, 64% of participants had average scores, and 21% had above average scores. The final knowledge test items and answers can be found in the Supplementary Material.

**Table 4:** Demographic variables, scores, means, and standard deviations for the knowledge test.

**Variable**	**Characteristic**	***N***	**Mean**	***SD***
Sex	Male	152	25.84	4.31
	Female	119	26.85	3.60
Age	18–29	54	24.80	4.96
	30–39	105	25.83	4.13
	40–49	42	26.57	3.29
	50–59	46	27.98	2.41
	60+	26	27.62	3.80
Race	White	218	26.59	3.91
	Black	22	24.14	3.54
	Native American	1	20.00	0.00
	Asian	21	25.52	5.33
	Biracial	5	27.60	1.82
	Other	7	25.20	5.17
Education	<Grade 12	2	19.00	4.24
	High school graduate	19	25.63	4.18
	Some college	38	26.76	3.12
	Associate degree	18	26.44	3.55
	University degree	123	26.28	4.32
	Graduate	70	26.63	3.81
Learning source	Internet	167	26.40	3.92
	Television	89	26.02	4.34
	Newspaper	8	24.50	3.16
	Friends	3	28.00	2.65
	Family	2	22.50	2.12
	Medical journals	3	31.00	1.73
	Work sources	1	30.00	0.00

## Discussion

A successful response to COVID-19 requires people around the world to understand evolving messages from governments and health authorities in order to protect themselves from infection and prevent disease spread. Government messaging has led to misunderstanding about the danger of COVID-19 (25), creating confusion and inaction (26). We developed a norm-referenced measure that can be used by health educators and researchers to better understand a layperson's knowledge of COVID-19 prior to the delivery of a health education program. If educators can interrupt and eliminate errors and misinformation, preventative measures will be more successful in reducing the spread of the virus.

The COVID-19 Knowledge Test assesses relevant medical terminology that has been cited in the news and in scientific journals. It includes questions concerning symptoms of the virus, relevant scientific discoveries, and pertinent findings that affect the safety of the general public. The test consists of 34 items that can be completed in <10 min. It also includes normative data that can be used by health educators to assess their clients' understanding of the disease.

We found strong evidence that the COVID-19 Knowledge Test is a unidimensional measure with acceptable split-half reliability. Analysis of the Rasch measurement model found that the test items range from easy to moderately difficult, and the total test information curve indicated that this is a good scale for discriminating between exceptionally low and average scores. Educators and researchers may use this test to make meaningful assessments of test takers' knowledge.

A limiting factor for this study was the span of available knowledge being spread on mainstream news channels and websites about COVID-19 due to the pandemic itself, possibly inflating normative data for this test. In the years ahead, it would be paramount to determine how much people learn about this disease and how prepared they are in the event of future outbreaks. A second limitation of the study is that some of the questions rely on current research. A year from now, those questions will need to be revised or deleted if the scientific knowledge of the diseases has changed. Future research is recommended to investigate the construct validity of the COVID-19 Knowledge Test in comparison with other health measures (e.g., 15-Item Health Knowledge Test) and to continue to develop normative data with a variety of groups.

## Data Availability Statement

The raw data supporting the conclusions of this article will be made available by the authors, without undue reservation.

## Ethics Statement

The studies involving human participants were reviewed and approved by New York University's Institutional Review Board. The participants provided their written informed consent to participate in this study.

## Author Contributions

LR collected the data, processed the statistical data, and drafted the manuscript. JB revised the final manuscript, finalized ethics approval, and assisted in summarizing the findings. All authors contributed to the article and approved the submitted version.

## Conflict of Interest

The authors declare that the research was conducted in the absence of any commercial or financial relationships that could be construed as a potential conflict of interest.
