# Supplementary material for: COVID-19 Knowledge Test: An Assessment Tool for Health Educators During the COVID-19 Pandemic
Source: Front Public Health. 2020 Nov 5;8:580204. doi: 10.3389/fpubh.2020.580204 (PMC7676894; doi:10.3389/fpubh.2020.580204)
Supplement: Supplementary file 1 [file Data_Sheet_1.docx]

COVID-19 Knowledge Test Items

This quiz examines your knowledge of COVID-19. Some questions are more difficult than others. Please select the most correct answer to the best of your knowledge.

1. What may predict severity of COVID-19 virus related symptoms^1^?

**a.** Viral load

b. Incubation period

2.  COVID-19 virus infects human cells through^2^

a. RFR-2 receptors.

**b.**ACE-2 receptors.

3. What is the range of incubation period for COVID-19 virus^3^?

a. 3 and 10 days

**b.** 1 and 14 days

4. Corona viruses are unique because of their^4^

a. Smooth surfaces

**b.** Crown-like spikes

5. An individual with difficulty breathing is tested for COVID-19 virus but the results come back clear and he is told he can go home. A few days later he dies from pneumonia and the doctors conclude he had COVID-19^5^. The is an example of:

**a.** A false negative

b. A true negative

6. What change of smell and taste is associated with COVID-19 virus symptoms^6^?

a. An increase

**b.** A decrease

7. Provides an indication of how well a virus is replicating in an infected person^7^.

a. Length of incubation time

**b.** Viral load

8. COVID-19 virus can spread^8^

**a.** When a person speaks closely to you

b. Only if a person coughs or sneezes on you

9. Patients with severe COVID-19 cases tend to have^1^

a. Shorter virus-shedding periods.

**b.** Longer virus-shedding periods.

10. COVID-19 virus remains active on cardboard for^9^

a. 12 hours

**b.** 24 hours

11. The MAIN symptoms of COVID-19 virus are^10^

a. runny nose, sore throat, and diarrhea

**b.** fever, dry cough, and fatigue

12. A test indicates the presence of a virus even though it was never actually present^5^. This is an example of:

**a.** A false positive

b. A false negative

13. One of the anti-malaria drugs first examined by the U.S. at the onset of the COVID-19 pandemic was^11^

**a.** Hydroxychloroquine

b. Tafenoquine

14. COVID-19 virus is most contagious from people with^12^

a. Zero symptoms

**b.** Symptoms

15. Which condition is a symptom of COVID-19 virus^10^?

a. Blotch inside the mouth

**b.** Diarrhea

16. Myalgia is defined best as^13^

**a.** Muscle pain

b. Fever

17. COVID-19 virus is sensitive to^14^

**a.** Ultraviolet light

b. Infrared light

18. The following drug may shorten recovery time for COVID-19 virus^10^

**a.** Remdesivir

b. Rocephin

19. Chest X-rays of COVID-19 virus pneumonia patients can be differentiated from classical viral pneumonia by^15^

a. blackness

**b.** ground glass opacities

20. If no mask is available what material would provide a better barrier for small droplets^16^.

**a.** plain weave cotton dishtowel

b. wool scarf

21. Which condition is a symptom of COVID-19^17^?

**a.** Reduced smell

b. Sudden feeling of heat

22. How many hours can COVID-19 virus remain on plastic^9^?

a. 23

**b.** 72

23. Coronaviruses can be stored for several years at^10^

a. A few degrees below freezing

**b.** -80 Celsius (-112 Fahrenheit)

24. The following disease is linked to COVID-19 virus^18^

a. Celiac disease

**b.** Diabetes

25. A major component of infection prevention for COVID-19 virus is^19^

a. Exercise

**b.** Surface decontamination

26. The period of time between the infection of an individual by a pathogen and the manifestation of the illness it causes^20^

a. Intubation

**b.** Incubation

27. In reference to COVID-19 what does the acronym PPE refer to^21^

**a.** Protective equipment such as masks, gloves, goggles

b. Polystyrene-Polyphenylene Elastic Coveralls

28. The Spanish flu is to H1N1 as COVID-19 is to^22^

**a.** SARS

b. EBOLA

29. Between 2000 and 2020 how many different corona virus infections have there been^23^?

a. 5

**b.** 3

30. The approximate survival time for critically ill COVID-19 virus patients is^24^

**a.** 1-2 weeks

b. 6-8 weeks

31. What population is the most vulnerable to severe cases of COVID-19 virus^25^?

**a.** Older age

b. Middle age

32. Asymptomatic people^26^

a. do not carry the disease

**b.** are potential sources of transmission

33. A medical term for when a virus replicates inside your body and is released into the environment^27^.

a. Viral expulsion

**b.** Viral shedding

34. HIV is to AIDS as SARS-CoV-2 is to^28^

a. SARS

**b.** COVID-19

*Bolded letters indicate the correct answer.

# References

1. Liu Y, Yan LM, Wan L, Xiang TX, Le A, Liu JM, Peiris M, Poon LL, Zhang W. Viral dynamics in mild and severe cases of COVID-19. *The Lancet Infectious Diseases,* 2020;*20*(6):656-7. doi:10.1016/S1473-3099(20)30232-2

2. Morley JE, & Vellas B. COVID-19 and older adult. *J Nutr Health Aging*. 2020;*24*(4):364-5. doi:10.1007/s12603-020-1349-9

3. Kumari T, Shukla V. Covid-19: Towards confronting an unprecedented pandemic. *International Journal of Biological Innovations*. 2020;*2*(1):1-10. doi:10.46505/IJBI.2020.2101

4. Cascella M, Rajnik M, Cuomo A, Dulebohn SC, Di Napoli R. StatPearls. Treasure Island (FL): StatPearls [Internet]; 2020. Features, Evaluation and Treatment Coronavirus (COVID-19)

5. Woloshin S, Patel N, Kesselheim AS. False Negative Tests for SARS-CoV-2 Infection – Challenges and Implications. *N Engl J Med.* 2020;383. doi:10.1056/NEJMp2015897

6. Kim GU, Kim MJ, Ra SH, Lee J, Bae S, Jung J, Kim SH. Clinical characteristics of asymptomatic and symptomatic patients with mild COVID-19. *Clinical Microbiology and Infection*. 2020;*26*(7):948.e1-e3. doi:10.1016/j.cmi.2020.04.040

7. Lakdawala S, Gaglia M. What we do and do not know about COVID-19’s infectious dose and viral loads. *The Conversation*. 2020. Available from:https://theconversation.com/what-we-do-and-do-not-know-about-covid-19s-infectious-dose-and-viral-load-135991

8. Shereen MA, Khan S, Kazmi A, Bashir N, Siddique R. COVID-19 infection: Origin, transmission, and characteristics of human coronaviruses. *Journal of Advanced Research*. 2020;24:91-8. doi:10.1016/j.jare.2020.03.005

9. Sarla, GS. COVID 19: Myths and Facts. *Research & Review: Management of Emergency and Trauma Nursing*. 2020; 2(2):5-8. doi:10.5281/zenodo.3742655

10. Yang P, Wang X. COVID-19: A new challenge for human beings. Cellular & Molecular Immonology. 2020;17:555-7. doi:10.1038/s41423-020-0407-x

11. Magagnoli  J﻿, Narendran  S﻿, Pereira  F﻿, et al. Outcomes of hydroxychloroquine usage in United States veterans hospitalized with Covid-19. medRxiv. Preprint posted April 23, 2020. doi:[10.1101/2020.04.16.20065920](http://dx.doi.org/10.1101/2020.04.16.20065920)

12. Amgain K, Neupane S, Panthi L, Thapaliya P. Myths versus Truths regarding the Novel Coronavirus Disease (COVID-2019) Outbreak. *Journal of Karnali Academy of Health Sciences*. 2020;*3*(1):1-6. Available from: http://jkahs.org.np/jkahs/index.php/jkahs/article/view/211

13. Gounden R, Blockman M. Statin-induced myopathy: Cardiovascular prescriber. *Cardiovascular Journal of Africa*. 2008;*19*(3):156-7. Available from: https://hdl.handle.net/10520/EJC23122

14. Cascella M, Rajnik M, Cuomo A, Dulebohn SC, Di Napoli R. StatPearls. Treasure Island (FL): StatPearls [Internet]; 2020. Features, Evaluation and Treatment Coronavirus (COVID-19)

15. Morley JE, & Vellas B. COVID-19 and older adult. *J Nutr Health Aging*. 2020;*24*(4):364-5. doi:10.1007/s12603-020-1349-9

16. Howard  J, Huang  A, Li  Z, et al. Face masks against COVID-19: an evidence review. *Preprints*. 2020;2020040203. doi:10.20944/preprints202004.0203.v1

17. Kim GU, Kim MJ, Ra SH, Lee J, Bae S, Jung J, et al. Clinical characteristics of asymptomatic and symptomatic patients with mild COVID-19. *Clinical Microbiology and Infection*. 2020;*26*(7):948.e1-e3. doi:10.1016/j.cmi.2020.04.040

18. Yang J, Zheng Y, Gou X, Pu K, Chen Z, Guo Q, et al. Prevalence of comorbidities and its effects in coronavirus disease 2019 patients: A systematic review and meta-analysis. International J of Infect Diseases. 2020;94:91–95. doi:10.1016/j.ijid.2020.03.017

19. Phua J, Weng L, Ling L, Egi M, Lim CM, Divatia, JV, et al. Intensive care management of coronavirus disease 2019 (SARS-CoV-2): Challenges and recommendations. *The Lancet Respiratory Medicine*. 2020;*8*(5):506–17. doi:10.1016/S2213-2600(20)30161-2

20. Merriam-Webster [Internet]. Incubation period[cited 2020 Aug 11]. Available from: www.merriam-webster.com/dictionary/incubation%20period

21. Repici A, Maselli R, Colombo M, Gabbiadini R, Spadaccini M, Anderloni A, et al. Coronavirus (COVID-19) outbreak: what the department of endoscopy should know. *Gastrointestinal Endoscopy*. 2020;*92*(1):192-97. doi:10.1016/j.gie.2020.03.019

22. Centers for Disease Control and Prevention. Types of Influenza Viruses [Internet]. 2019. Available from: www.cdc.gov/flu/about/viruses/types.htm

23. LeDuc JW & Barry MA. SARS, the First Pandemic of the 21st Century. *Emerging Infectious Diseases*. 2004;*10*(11):e26. doi:10.3201/eid1011.040797_02

24. Yang X, Yu Y, Xu J, Shu H, Liu H, Wu Y, et al. Clinical course and outcomes of critically ill patients with SARS-CoV-2 pneumonia in Wuhan, China: A single-centered, retrospective, observational study. *The Lancet Respiratory Medicine*. 2020;*8*(5):475-481. doi:10.1016/S2213-2600(20)30079-5

25. Jordan RE, Adab P, & Cheng KK. SARS-CoV-2: Risk factors for severe disease and death. *BMJ*. 2020;368:m1198. doi:10.1136/bmj.m1198

26. Rothe C, Schunk M, Sothmann P, Bretzel G, Froeschl G, Wallrauch C, et al. Transmission of 2019-nCoV infection from an asymptomatic contact in Germany.  *New England Journal of Medicine*. 2020;*382*(10):970-1. doi:10.1056/NEJMc2001468

27. WebMD. What is virus shedding [Internet]? 2020. Available from: www.webmd.com/lung/qa/what-is-viral-shedding

28. World Health Organization. Naming the coronavirus disease (COVID-19) and the virus that causes it [Internet]. 2020. Available from: https://www.who.int
